# Dereplication of Natural Products from an Endophytic Fungus from *Ocotea diospyrifolia* with Anti-Inflammatory Potential Through Dual Modulation of PGE_2_ and LTB_4_ Release

**DOI:** 10.3390/molecules31193499

**Published:** 2026-10-01

**Authors:** Giovanni S. Oliveira, Marcus V. A. Marques, Ana L. U. Nascimento, Valmore H. P. Santos, Daniela A. Garcia, Lara P. D. M. Costa, Thiago A. M. Brito, Josean F. Tavares, Jorge M. David, Daniela A. Chagas-Paula, Eliane O. Silva

**Affiliations:** 1 Department of Organic Chemistry, Institute of Chemistry, Federal University of Bahia, Salvador 40170-115, BA, Brazil; giovanni.santos@ufba.br (G.S.O.); marcusmarques@ufba.br (M.V.A.M.); analuisa.urquiza@gmail.com (A.L.U.N.); valmorehenrique@ifba.edu.br (V.H.P.S.); jmdavid@ufba.br (J.M.D.); 2 Institute of Chemistry, Federal University of Alfenas, Alfenas 37130-001, MG, Brazildaniela.chagas@ufjf.br (D.A.C.-P.); 3 Institute for Research in Pharmaceuticals and Medications, Federal University of Paraíba, Campus I, João Pessoa 58051-900, PB, Brazil; thiagobrito@ltf.ufpb.br (T.A.M.B.); josean@ltf.ufpb.br (J.F.T.); 4 Department of Chemistry, Federal University of Juiz de Fora, Juiz de Fora 36036-900, MG, Brazil

**Keywords:** endophytic fungi, anti-inflammatory natural products, dual PGE_2_/LTB_4_ inhibition, comparative metabolomics, UHPLC-HRMS/MS

## Abstract

Endophytic fungi are significant sources of structurally diverse natural products with promising pharmacological and biological applications. In this study, endophytic fungi isolated from *Ocotea diospyrifolia* leaves were investigated as potential producers of anti-inflammatory natural products using an integrated workflow combining biological screening, comparative metabolomics, and high-resolution mass spectrometry-based dereplication. Crude extracts from five endophytic fungi (designated as OD1, OD5, OD6, OD7, and OD8) were screened for their ability to inhibit prostaglandin E_2_ (PGE_2_) and leukotriene B_4_ (LTB_4_) release. *Lentinus* sp. OD5 extract displayed promising dual inhibitory activity, and its UHPLC-QTOF-HRMS-based metabolomic profiling enabled the annotation of two compounds. Mass spectrometry data analysis, combined with SIRIUS annotation, putatively identified rhodojaponin, a grayanane diterpene with reported anti-inflammatory activity, and tinocordiside, an immunomodulatory diterpenoid glycoside. These findings expand the chemical diversity currently associated with endophytic fungi and provide a strong foundation for the future isolation, structural confirmation, and pharmacological evaluation of anti-inflammatory metabolites with potential therapeutic applications.

## 1. Introduction

The inflammatory process is a crucial biological defense mechanism against harmful stimuli, playing an essential role in tissue repair and host protection. However, when dysregulated or sustained, inflammation can contribute to the onset and progression of various pathological conditions, including osteoarthritis, diabetes mellitus, neurodegenerative disorders such as Alzheimer’s and Parkinson’s diseases, cardiovascular diseases, and obesity [1,2]. The onset and progression of inflammation are largely driven by the overproduction of lipid mediators derived from arachidonic acid generated through the cyclooxygenase (COX) and lipoxygenase (LOX) pathways [3]. The COX enzymes comprise COX-1 and COX-2 isoforms, which catalyze the biosynthesis of prostaglandins, whereas LOXs are responsible for the formation of leukotrienes and other bioactive lipid mediators involved in the inflammatory response [4,5].

Traditional nonsteroidal anti-inflammatory (NSAID) drugs exert their therapeutic effects through non-selective inhibition of COX. The result is gastrointestinal injury due to constitutive COX-1 inhibition, while diversion of arachidonic acid metabolism toward the LOX pathway may increase leukotriene production, contributing to hypersensitivity reactions and bronchospasm [3]. Selective COX-2 inhibitors, known as ‘coxibs’, were developed to reduce gastrointestinal toxicity but have been associated with an increased risk of adverse cardiovascular events [6,7]. In this context, there is a pressing need to develop novel anti-inflammatory agents capable of simultaneously suppressing the biosynthesis of both prostaglandins and leukotrienes. Dual COX/LOX inhibitors, particularly those targeting COX and 5-LOX, suppress the production of key pro-inflammatory mediators and represent a rational strategy for developing safer and more effective anti-inflammatory therapies [7]. Beyond inflammatory disorders, the concept of dual COX/5-LOX inhibition has also gained increasing attention in the treatment of several pathological conditions, including cancer [8] and neurodegenerative disorders like Alzheimer’s and Parkinson’s disease [9,10].

Natural products remain one of the richest sources of structurally diverse bioactive molecules and have yielded numerous compounds with promising anti-inflammatory properties [11,12]. Several classes of natural products, such as alkaloids, flavonoids, terpenoids, iridoids, and coumarins, have been identified as dual inhibitors of COX and LOX enzymes [13]. The genus *Ocotea* (Lauraceae) encompasses approximately 400 species, many of which are native to South America and have long been investigated for their broad pharmacological potential, particularly in the treatment of rheumatic disorders [14]. Metabolomics and phytochemical studies have shown that *Ocotea* species are valuable sources for bioprospecting anti-inflammatory compounds [15]. The anti-inflammatory properties of *O. odorifera* [16] and *O. diospyrifolia* have been reported, including the identification of its main active compounds able to inhibit prostaglandins and/or leukotrienes [17].

Endophytic fungi inhabit internal plant tissues without causing any harm because they usually establish mutualistic associations with their hosts, promoting plant growth and increasing tolerance to biotic and abiotic stresses [18]. The relationship between medicinal plants and their endophytic communities is particularly attractive from a bioprospecting perspective. Host plants with well-established pharmacological properties have been recognized as reservoirs for microorganisms capable of producing bioactive metabolites that are structurally related to, or even identical to, those found in their host plants [19]. These phenomena have stimulated extensive research into endophytic microorganisms as sustainable and alternative sources of valuable bioactive natural products, thereby reducing pressure on medicinal plant populations.

The ability of endophytic fungi to produce specialized metabolites, which were originally thought to be unique to their host plants, has been explained by several hypotheses. These include horizontal gene transfer during long-term plant–fungus interactions, adaptive responses to shared environmental cues (xenohormesis), and the independent evolution of similar biosynthetic pathways under similar selective pressures [20]. Despite the increasing current interest in chemistry and pharmacological properties of *Ocotea* species [21], little is known about the anti-inflammatory potential of their associated endophytic fungi. Furthermore, studies combining bioactivity assessment with comprehensive metabolomic characterization of these microorganisms remain limited.

The present study describes the anti-inflammatory potential of extracts from endophytic fungi isolated from *O. diospyrifolia* and characterizes their chemical profiles using ultra-high-performance liquid chromatography coupled to high-resolution mass spectrometry (UHPLC-HRMS). By integrating biological screening with untargeted metabolite profiling, we identified promising bioactive fungal extracts and putatively annotated metabolites associated with their chemical profiles, providing new insights into the chemical diversity and anti-inflammatory potential of *Ocotea*-associated endophytic fungi.

## 2. Results

### 2.1. Screening of Endophytic Fungal Extracts for Dual Modulation of PGE2 and LTB4 Release

The protocol employed for the isolation of endophytic fungi from *O. diospyrifolia* leaves yielded five strains designated OD1, OD5, OD6, OD7, and OD8. Prior to the endophyte identification, each strain was cultivated in potato dextrose broth (PDB) for 10 days. After this period, their metabolites were extracted using ethyl acetate (EtOAc). All extracts were then analysed by an ex vivo anti-inflammatory assay in human whole blood, which measured the prostaglandin E_2_ (PGE_2_) and leukotriene B_4_ (LTB_4_) levels.

**Figure 1:**
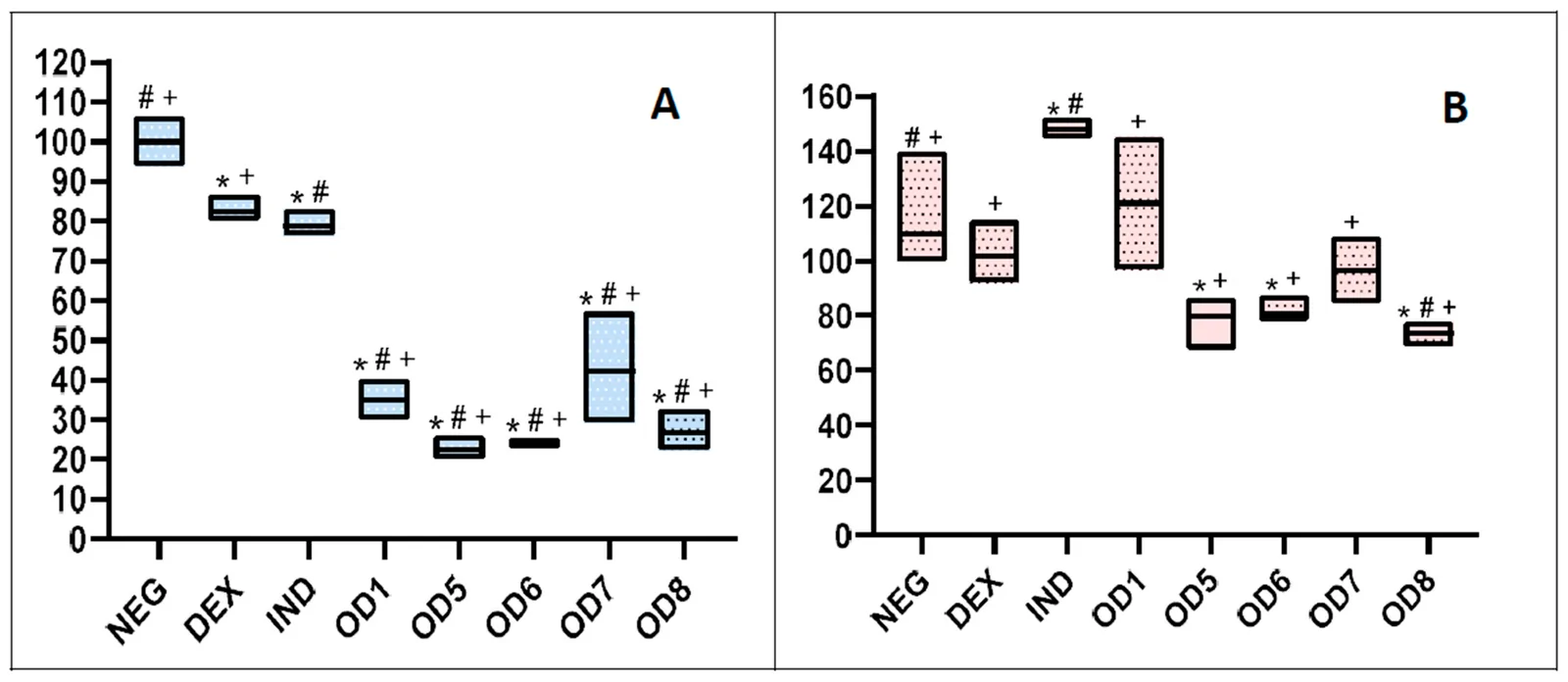
Anti-inflammatory activity of endophytic fungal extracts evaluated through inhibition assays of (**A**) PGE_2_ and (**B**) LTB_4_ pathways. Dexamethasone (DEX) and indomethacin (IND) were used as reference anti-inflammatory drugs, while the negative control (NEG) consisted of treatment with vehicle. The assay was conducted in three authentic experimental replicates (*n* = 3) and analyzed by one-way ANOVA and Dunnett’s multiple comparisons post-test. The bars represent mean ± standard deviation (SD). Asterisks (*) denote a statistically significant difference compared to the negative control (*p* ≤ 0.05). Symbols # and + indicate a significant difference (*p* ≤ 0.05) compared to DEX or IND at a concentration of 1 μg/mL, respectively.

The assays demonstrated remarkable inhibitory activity against the production of both PGE_2_ and LTB_4_ by the evaluated fungal extracts (Figure 1). This suggests that these extracts contain specialized metabolites that act as dual inhibitors of PGE_2_ and LTB_4_ release. Among the fungal extracts, those designated OD5, OD6, and OD8 showed the strongest inhibitory effects, markedly reducing PGE_2_ levels compared to the negative control (*p* < 0.05). In contrast, the OD1 and OD7 extracts exhibited lower activity. The most pronounced decrease in LTB_4_ levels was observed with the OD5, OD6, and OD8 extracts, while the OD1 and OD7 extracts were inactive and did not show a statistically significant difference from the negative control. Overall, the fungal extracts OD5, OD6, and OD8 exhibited a concomitant inhibitory effect on the release of the pro-inflammatory mediators PGE_2_ and LTB_4_. Among these extracts, OD5 was selected for subsequent genus identification, chemical characterization, and metabolic profile analysis.

### 2.2. Comparative Metabolite Profiling of the Endophytic Fungi

**Figure 2:**
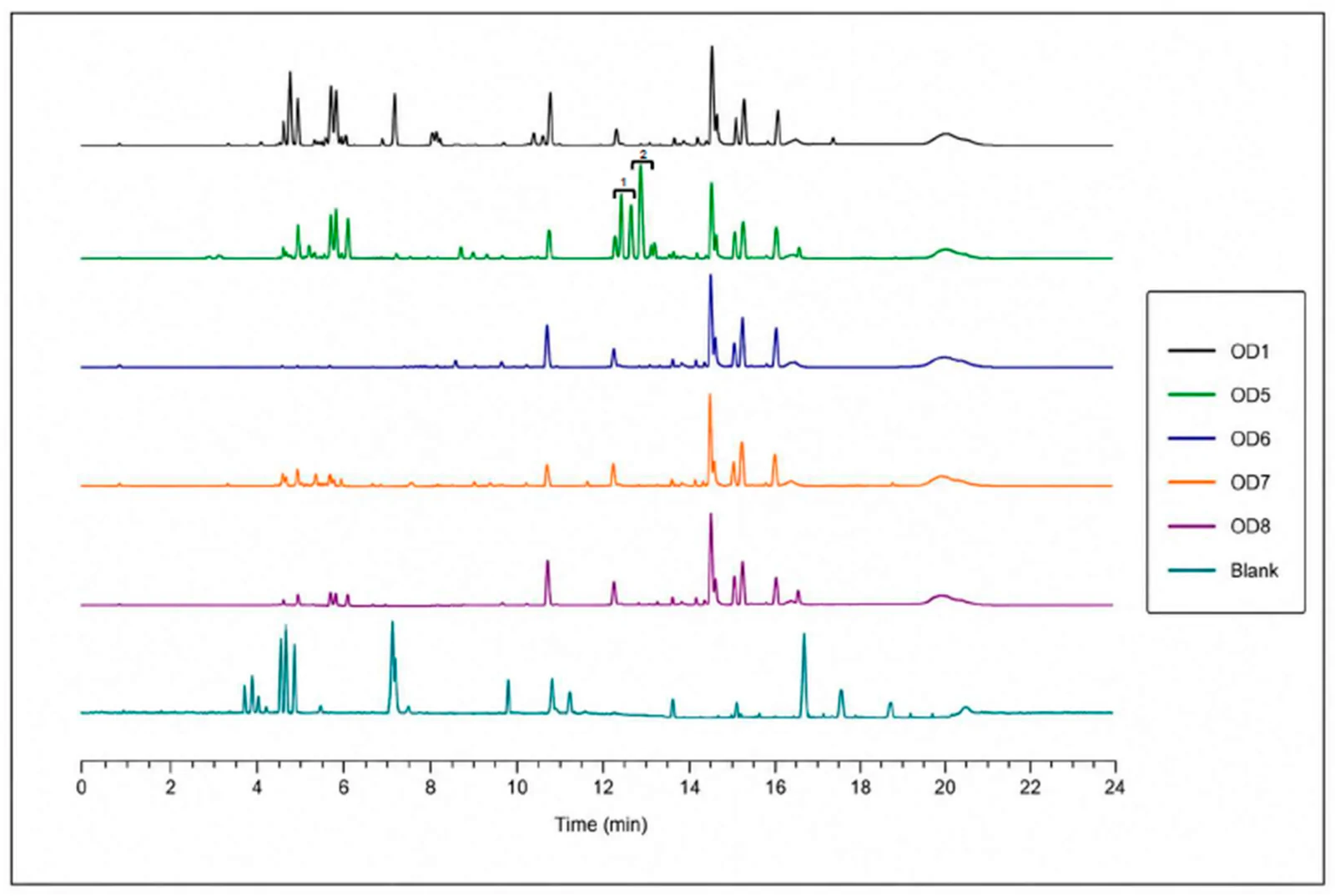
UHPLC-HRMS total ion chromatograms (TICs) of the ethyl acetate extracts obtained from endophytic fungal strains OD1, OD5, OD6, OD7, and OD8. The blank sample corresponds to an ethyl acetate extract of uninoculated culture medium processed under the same conditions. In the OD5 extract (green TIC), three metabolites were putatively identified: two isomeric compounds (**1**) at *m*/*z* 419.2040, detected at 12.70 and 12.95 min; compound **2** at *m*/*z* 433.2195, detected at 13.16 min.

Total ion chromatograms (TICs) acquired by UHPLC-HRMS (Figure 2) from the EtOAc extracts of endophytic fungi strains OD1, OD5, OD6, OD7, and OD8 allowed for a comparative analysis of their specialized metabolite profiles. A visual inspection of the TICs revealed metabolic variation among the endophytes. Notably, the OD5 strain exhibited a distinct cluster of metabolites that eluted between 12.5 and 13.5 min, which was not observed in the other endophyte extracts. The presence of these unique chromatographic features suggests that OD5 produces strain-specific specialized metabolites, potentially contributing to the promising anti-inflammatory activity associated with this strain.

### 2.3. Phylogenetic OD5 Identification

**Figure 3:**
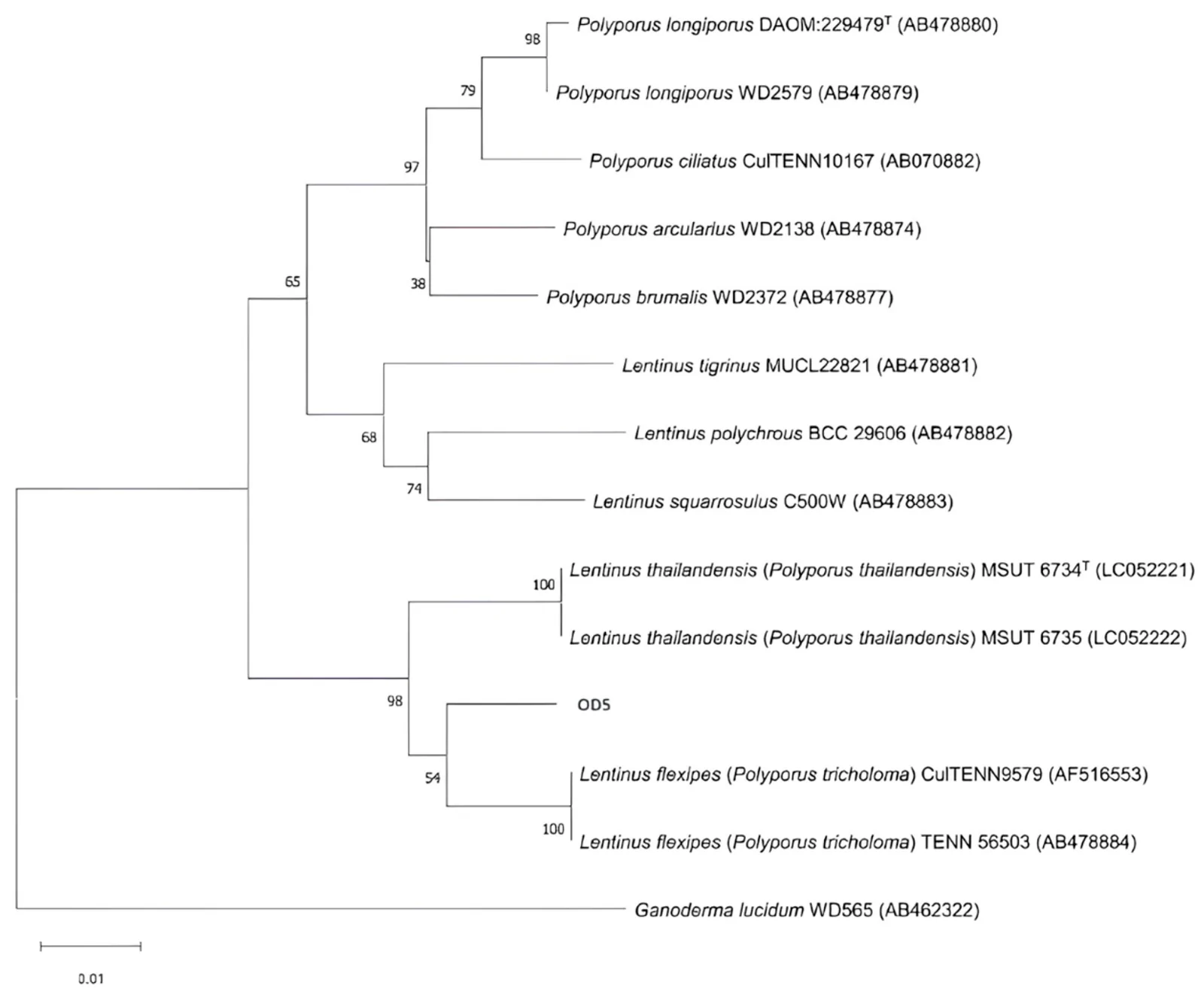
Phylogenetic placement of *Lentinus* sp. for endophytic fungus OD5 based on its ITS sequencing. The achieved DNA sequence was compared with the reference sequences deposited in GenBank. Phylogenetic trees were constructed using the Neighbor-Joining method in MEGA 6. Bootstrap analysis was performed with 1000 replicates, and support values are indicated at the nodes. Scale bars represent 0.01 substitutions per nucleotide site.

Phylogenetic analysis based on the internal transcribed spacer (ITS) region supported the placement of the OD5 among closely related taxa, as shown in the phylogenetic tree (Figure 3). Sequence similarity searches revealed that the ITS sequence of OD5 shared 95–99% identity with sequences assigned to *Lentinus flexipes* (formerly *Polyporus tricholoma*), deposited in the GenBank database. The node linking OD5 to the *L. flexipes* clade exhibited moderate-to-low bootstrap support (54%). In contrast, the broader clade comprising OD5 and *L. flexipes* was strongly supported (98%), confirming its close phylogenetic affiliation within *Lentinus* genus. While the ITS sequence similarity and phylogenetic structure support the classification of OD5 as *Lentinus*, the relatively low bootstrap value at the OD5-*L. flexipes* node does not provide enough backing for a confident species-level identification. Therefore, the combined evidence from sequence similarity searches and phylogenetic analysis supports identifying strain OD5 as *Lentinus* sp.

### 2.4. Putative Annotation of Lentinus *sp.* OD5-Specific Metabolites via UHPLC-HRMS/MS

Considering the dual inhibition of both PGE_2_ and LTB_4_ release displayed by *Lentinus* sp. OD5 extract, along with its unique metabolic profile, it became the primary target for HRMS/MS annotation after chromatographic separation. To refine the analysis, an integrated MZmine-SIRIUS platform analysis was performed to putatively characterize the main natural products detected in *Lentinus* sp. OD5 extract.

MZmine feature detection and alignment (Table S1, Supplementary Materials) confirmed the presence of three features in the extract of *Lentinus* sp. OD5. These features exceed the established detection threshold and were not observed in the other strains. They eluted within a narrow chromatographic window between 12.50 and 13.50 min, comprising two isomeric ions at *m*/*z* 419.2040 (**1**, RT 12.70 and 12.95 min) and a third compound at *m*/*z* 433.2197 (**2**, RT 13.16 min).

**Figure 4:**
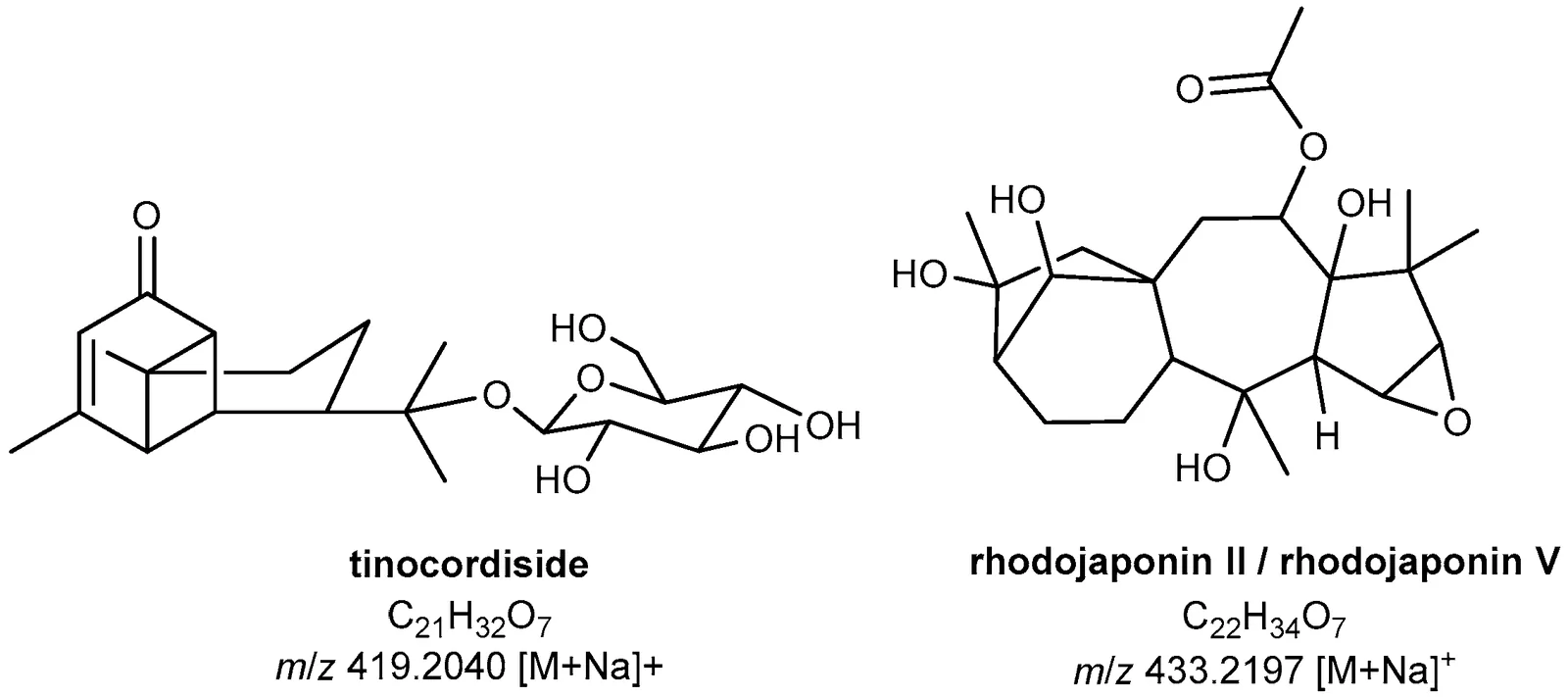
Chemical structures, molecular formulas, and accurate mass data of tinocordiside (**1**) and rhodojaponin II/V (**2**), putatively identified based on HRMS/MS data and SIRIUS analysis. The annotations were assigned at Level 2 confidence. The stereochemistries of both compounds remained undetermined.

Dereplication of metabolites at *m*/*z* 419.2040 and 433.2197 was performed using LC-MS/MS data analysis, computational analysis via the SIRIUS platform, and the proposal of manual fragmentation pathways. Metabolite annotations were reported at confidence level 2 according to the criteria proposed by the Metabolomics Standards Initiative [22]. For each detected feature, candidate molecular structures were predicted using SIRIUS by evaluating the acquired MS2 data against computationally generated fragmentation information. Candidate prioritization considered both the resulting spectral similarity scores and manual inspection of diagnostic product ions, as well as their corresponding fragmentation pathways. The most plausible structural assignments were subsequently verified through searches in the SciFinder database. Based on this workflow, specialized metabolites **1** and **2** were detected in *Lentinus* sp. OD5 extracts were annotated (Table 1 and Figure 4).

**Figure 5:**
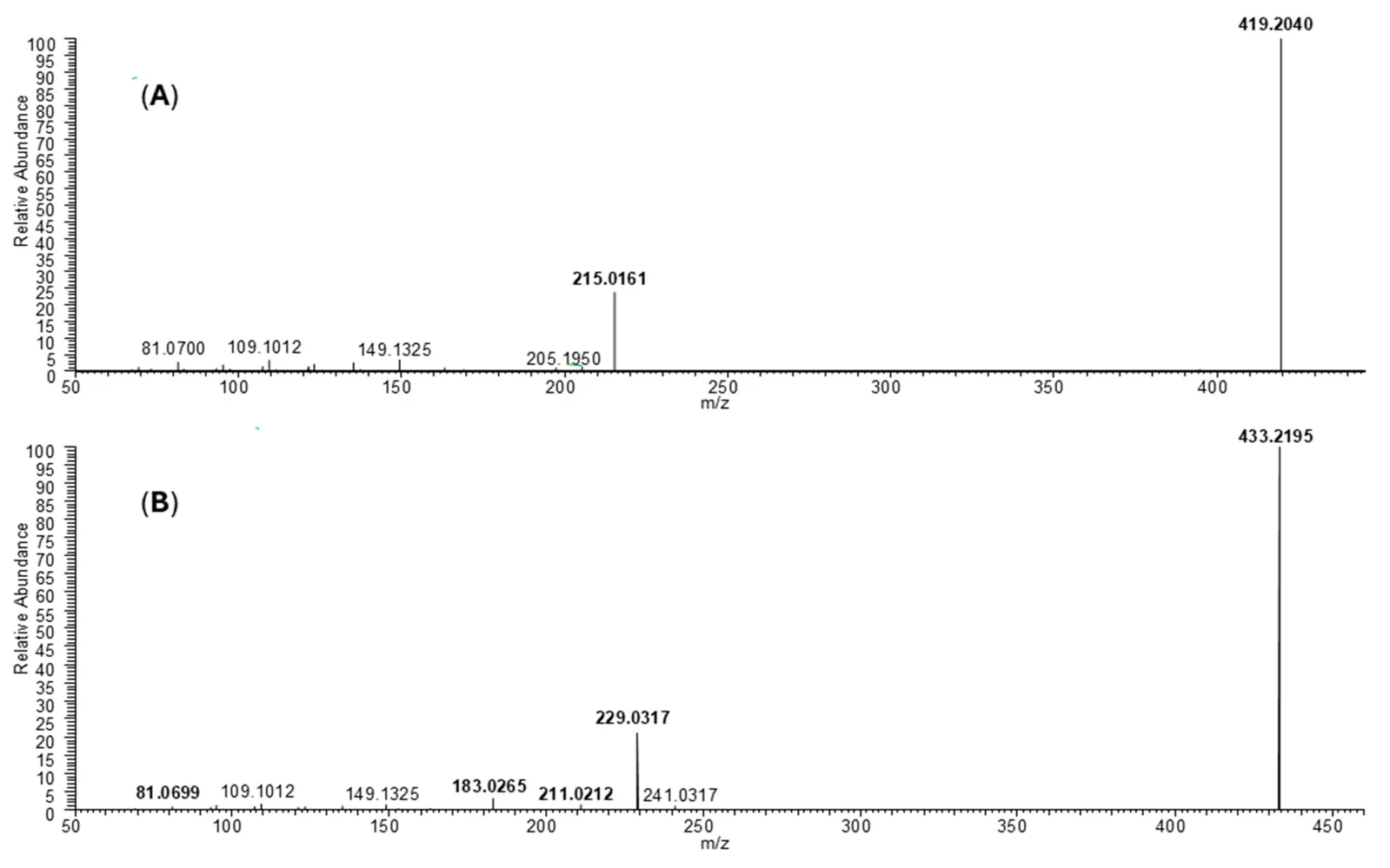
MS2 ESI-(+)-QTOF spectra for tinocordiside ((**A**) *m*/*z* 419.2040) and rhodojaponin II/V ((**B**) *m*/*z* 433.2197). The diagnostic fragment ions proposed in the fragmentation pathways (Figure 6) are shown in bold in the spectra and support the tentative identification of both compounds.

Two chromatographically separated metabolites exhibited identical precursor ions at *m*/*z* 419.2040 ([M+Na]^+^), indicating the occurrence of isomeric compounds, as both generated essentially identical MS2 spectra. SIRIUS annotation assigned such compounds as tinocordiside (**1**) and/or related isomeric structures. Additionally, HRMS data for the precursor ion detected at *m*/*z* 433.2197 ([M+Na]^+^) showed an excellent mass accuracy (Δ - 0.46 ppm), allowing reliable elemental composition prediction by SIRIUS software. The predicted molecular formula was C_22_H_34_O_7_, matching the diterpenoid rhodojaponin II or rhodojaponin V (**2**). SIRIUS ranked rhodojaponin II as the highest-scoring candidate in its natural products database, supporting this tentative annotation. However, rhodojaponin V shares the same molecular formula (C_22_H_34_O_7_) and has distinct stereochemistry [23] making it a viable alternative structural candidate that cannot be disregarded based solely on the available MS data. The chemical structures of **1** and **2**, along with their MS2 spectra, are depicted in Figure 4 and Figure 5, respectively.

**Figure 6:**
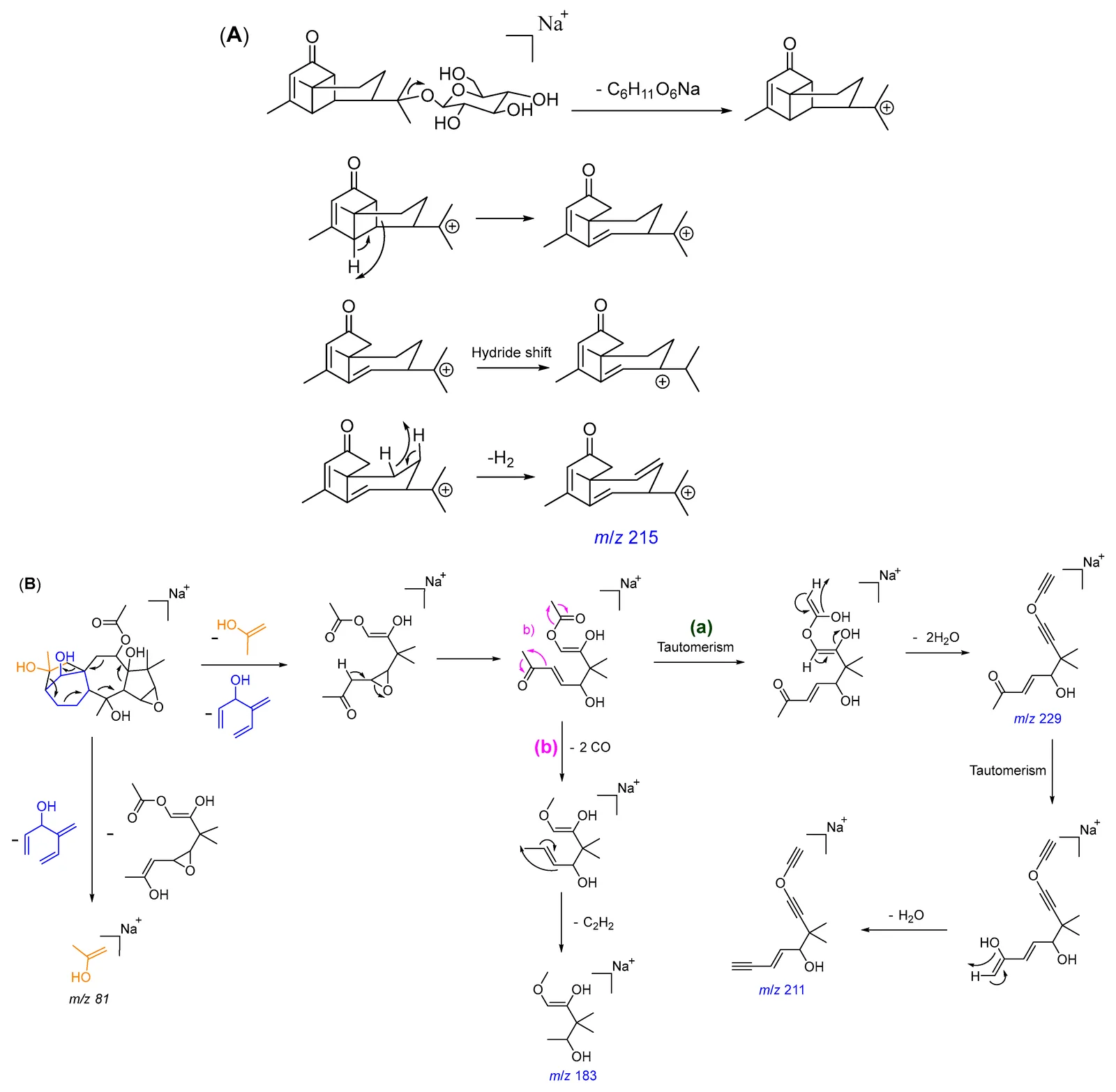
Proposed fragmentation pathway of tinocordiside ((**A**) *m*/*z* 419.2040) and rhodojaponin II/V ((**B**) *m*/*z* 433.2197) in ESI-(+)-QTOF.

Manual curation of each MS2 spectrum was performed to improve the reliability of compound annotation. Therefore, the fragmentation patterns obtained from HRMS were carefully analyzed and proposed (Figure 6). The MS2 spectrum (Figure 5A) of tinocordiside (**1**) displayed a base peak at *m*/*z* 419.2040 [M+Na]^+^, consistent with the molecular formula C_21_H_32_O_7_. Basically, this ion generated a single fragment at *m*/*z* 215, resulting from loss of the sodium glycosyl moiety, followed by a hydride shift, carbocation rearrangement, and a neutral loss of H_2_ (Δ*m*/*z* = 204 Da; Figure 6A). The resulting carbocation is stabilized by conjugation with two double bonds.

Rhodojaponin II (**2**) exhibited a base peak at *m*/*z* 433.2195 ([M+Na]^+^, C_22_H_34_O_7_) in the MS2 spectrum (Figure 5B). Retro-Dials-Alder and other successive ring cleavages of the **2** may lead to the positive fragment observed at *m*/*z* 81. On the other hand, ions at *m*/*z* 183, 211, and 229 provide additional evidence of parallel dissociation routes that may involve tautomerism, loss of CO and C_2_H_2_, or dehydration (Figure 6B).

## 3. Discussion

The ex vivo bioassay screening revealed remarkable anti-inflammatory potential in the EtOAc extracts of five endophytic fungi isolated from *O. diospyrifolia*, a well-documented plant species as a source of anti-inflammatory natural products [17]. The fungal extracts displayed distinct anti-inflammatory profiles against PGE_2_ and LTB_4_ releases (Figure 1). Under the conditions evaluated, all extracts showed significant anti-inflammatory activities. The varying inhibitory profiles observed suggest that the fungal extracts may contain chemically diverse specialized metabolites responsible for the activities. Additionally, the presence of a metabolite or a combination of metabolites (synergistic effects) can exert broader anti-inflammatory effects. The pronounced decrease in PGE_2_ levels observed in the OD5, OD6, and OD8 endophyte extracts, along with their notable ability to reduce LTB_4_ levels, is particularly relevant. Natural products with dual activity toward both PGE_2_ and LTB_4_ have been investigated as potential anti-inflammatory leads, underscoring the pharmacological relevance of compounds capable of modulating these important inflammatory mediators [16,17,24]. The release of PGE_2_ is one of the main targets of anti-inflammatory drugs, with a key role in a wide range of inflammatory diseases that rely on the COX activation cascade [3]. The results described herein may be directly related to the COX enzyme, upstream in the inflammatory cascade, through the COX pathway, or at other locations. Therefore, given the promising results, the precise mechanism of action warrants further evaluation. Additionally, a cytotoxicity assessment was not performed for the fungal extracts, which represents a limitation of the present study.

The pronounced anti-inflammatory activity observed in the evaluated fungal extracts is consistent with previous reports. For example, ergosterol derivatives isolated from the endophytic fungus *Fusarium* sp. exhibited significant inhibition of 5-LOX [25], and specialized metabolites from the endophyte *Penicillium chrysogenum* displayed significant inhibition of COX-2 with IC_50_ values in the submicromolar to low micromolar range [26]. Similarly to the hypothesis underlying our study, the anti-inflammatory potential of endophytic fungi isolated from *Elaeocarpus floribundus* Blume was investigated, considering the long-standing use of this plant in traditional medicine for the treatment of rheumatoid arthritis. As a result, COX and 5-LOX inhibitory assays state that the *Aspergillus niger* fraction possesses the greatest activity against these enzymes that are responsible for producing PGE_2_ and LTB_4_, respectively [27].

An additional relevant aspect of our results is that the EtOAc extracts were obtained from endophytic fungi cultivated under identical conditions. Therefore, the differences in bioactivity observed among extracts may reflect intrinsic differences in their biosynthetic capacities. The significant differences in the inhibition of PGE_2_ and LTB_4_ among the five isolates suggest considerable strain-dependent pharmacological diversity. Among the evaluated endophytes, OD5 was considered as particularly promising due to its impressive ability to simultaneously inhibit the release of both PGE_2_ and LTB_4_, along with its unique chemical profile. This concurrent activity provided a strong rationale for prioritizing it for UHPLC-HRMS/MS-based metabolomic investigation and annotation of its main bioactive metabolites.

Phylogenetic analysis based on the ITS region placed strain OD5 within the *Lentinus* lineage with 95–99% ITS similarity, supporting its assignment to this genus (Figure 3). The use of a single barcode locus may underestimate cryptic diversity or fail to distinguish closely related species. Although the ITS region is widely used as the primary fungal DNA barcode because of its broad taxonomic coverage and practical application, its discriminatory power is not uniform across fungal lineages [28]. Closely related species may share highly similar ITS sequences, resulting in poorly resolved or weakly supported species-level relationships. Therefore, the clustering of OD5 near *Lentinus flexipes* should be interpreted as evidence of a close phylogenetic relationship rather than as conclusive evidence for species-level identification. Phylogenetic reconstruction further placed OD5 within the *Lentinus* clade, although species-level resolution was not achieved due to the limited discriminatory power of the ITS marker within this group.

Species of *Lentinus* are widely distributed and have attracted considerable interest because of their ecological versatility and capacity to produce structurally diverse specialized metabolites. In many Asian countries, *Lentinus* species are not only valued as traditional medicinal resources but are also widely consumed as edible mushrooms [29]. Numerous chemical and pharmacological studies have demonstrated that *Lentinus* spp. exhibit a broad range of beneficial biological activities, including anti-inflammatory effects [30]. In our studies, the OD5 strain was obtained as an endophytic fungus from *O. diospyrifolia*, rather than isolating it from a free-living basidiome. That is especially interesting because endophytic colonization may expose fungi to host-associated ecological pressures, which could influence the biosynthesis of specialized metabolites.

Recent advancements in chemical dereplication and database informatics have fundamentally changed the study of bioactive natural products and significantly accelerated the lead discovery process [31,32]. These approaches allow for the rapid characterization of specialized metabolites and facilitate the correlation between chemical composition and biological activity, thereby accelerating the selection of promising sources of bioactive compounds.

The comparison of TICs obtained by UHPLC-QTOF-HRMS (Figure 2) confirmed substantial differences in the chemical profiles of the five endophytic fungal extracts. Notably, the TIC profile of the *Lentinus* sp. OD5 extract exhibited a distinct cluster of chromatographic signals eluting between approximately 12 and 14 min, which was not detected in the profiles of the other endophytic fungal extracts. The strain-dependent chemical diversity, along with the results from the anti-inflammatory screening, prompted a comparative metabolomic analysis. The detection of certain metabolites associated with *Lentinus* sp. OD5 may indicate strain-specific variations in the expression or regulation of biosynthetic pathways that are either absent, weakly expressed, or differently regulated in the other endophytic fungi examined. Chemically distinctive extracts exhibiting relevant biological activity therefore represent compelling targets for dereplication and subsequent chemical structure annotation. In this context, *Lentinus* sp. OD5 extract emerged as a priority due to its pronounced anti-inflammatory activity and distinctive chemical profile. Accordingly, comparative metabolomics, feature extraction, and dereplication were subsequently employed to tentatively identify metabolites uniquely associated with *Lentinus* sp. OD5 and prioritize candidate compounds for further investigation.

Putative identification of metabolites in complex samples remains a significant challenge in natural products research and untargeted metabolomics. HRMS has been widely used for metabolite characterization due to its high sensitivity and high-throughput capability. Several computational approaches have been developed to infer molecular classes from mass data [33]. SIRIUS uses precursor and fragment information to generate molecular formulas and fragmentation trees, ranking candidate structures by their compatibility with experimental MS2 data [34]. It uses structural information encoded in experimental MS2 spectra to predict the chemical classes of unknown metabolites. Its predictions are based on spectral features learned from millions of reference compounds, enabling the characterization of metabolites even when their exact structures cannot be determined.

MZmine’s feature detection and alignment confirmed the existence of three molecular features that were detected exclusively in the *Lentinus* sp. OD5 extract. These features were identified above the feature-detection threshold set during data processing and eluted within a narrow chromatographic window between 12.50 and 13.50 min (Table 1). These features included two chromatographically separated ions at *m*/*z* 419.2040 and one ion at *m*/*z* 433.2197. The annotation workflow combined accurate precursor mass analysis, MS2 fragmentation, in silico spectral interpretation using SIRIUS, and database cross-referencing, in addition to manual curation of fragment assignments. Such an integrative approach aligns with current metabolomics practice, where metabolite annotation is based on a body-of-evidence strategy rather than on accurate mass alone [31].

The two chromatographically separated features at *m*/*z* 419.2040 exhibited identical nominal precursor ions and MS2 fragmentation patterns (Figure 5A), consistent with the occurrence of isomeric forms. The observed accurate mass is consistent with the sodium adduct ([M+Na]^+^) of a metabolite with molecular formula C_21_H_32_O_7_, corresponding to a neutral monoisotopic mass of 396.2148 Da. This formula has been annotated as tinocordiside (**1**), a rearranged cadinane sesquiterpene glycoside originally isolated from the medicinal plant *Tinospora cordifolia* [35]. The definitive identification of this compound, including the determination of its stereochemistry, will require spectroscopic techniques, particularly nuclear magnetic resonance (NMR). The biological relevance of the annotation of compound **1** at a confidence level of 2 is noteworthy and corroborates our results. This compound was originally isolated from an immunomodulatory fraction of *T. cordifolia*, establishing an early connection between this metabolite and its role in modulation of biological processes related to inflammation and immune responses [35].

Finally, the feature detected at *m*/*z* 433.2197 was also particularly informative. The measured mass error of −0.46 ppm provided strong support for the elemental composition C_22_H_34_O_7_. This molecular formula corresponds to rhodojaponin II or V (**2**), which are highly oxygenated grayanane diterpenoids. SIRIUS ranked **2** among the highest-scoring candidates in its natural-products database. Additionally, the agreement between the accurate precursor mass, the predicted molecular formula, the database candidate, and the observed MS2 fragmentation (Figure 6) collectively support its putative annotation. Rhodojaponin II (**2**) is one of the most abundant diterpenoids isolated from both roots and leaves of *Rhododendron molle* G. Don (Ericaceae) [36]. Compound **2** has previously been investigated in inflammatory responses, and its ability to inhibit TNF-α-induced secretion of inflammatory cytokines in human rheumatoid arthritis fibroblast-like synoviocytes has been reported [37].

## 4. Materials and Methods

### 4.1. Culture Media, Solvents, and Reactants

Potato Dextrose Agar (PDA; Cat. No. K25-1022) and Potato Dextrose Broth (PDB; Cat. No. K25-1261) were purchased from Kasvi (Pinhais, PR, Brazil). Ethyl acetate (EtOAc; P.A./ACS grade; Cat. No. AE09827RA), ethanol (EtOH; 96%; P.A./ACS grade; Cat. No. AE07321RA), formic acid (P.A./ACS grade; Cat. No. AF09029RA), and acetic acid (AcOH; P.A./ACS grade; Cat. No. AA09870RA) were purchased from Êxodo Científica (Sumaré, SP, Brazil). Methanol (MeOH; HPLC grade; Cat. No. 1.06007.4000) and acetonitrile (ACN; HPLC grade; Cat. No. 100030) were purchased from Merck (Darmstadt, HE, Germany). Ultrapure water was obtained using a Milli-Q^®^ Direct 8 water purification system (Merck Millipore, Burlington, MA, USA).

Dexamethasone (≥97%; Cat. No. D4902) and indomethacin (98.0–102.0%; Cat. No. I7378), Limaprost (≥98%; Cat. No. SML1532) and lipopolysaccharide (LPS) were also purchased from Sigma-Aldrich (St. Louis, MO, USA).

LC-18 solid-phase extraction (SPE) cartridges (Supelclean™ LC-18 SPE Tubes; Cat. No. 57054) were purchased from Supelco/Sigma-Aldrich (Bellefonte, PA, USA). Sterile 96-well microplates (clear, flat-bottom; Cat. No. 3596) were obtained from Corning Inc. (Corning, NY, USA).

Glass microspheres (425–600 μm), used for fungal genomic DNA extraction, were purchased from Sigma-Aldrich (St. Louis, MO, USA). The BigDye Terminator v3.1 Cycle Sequencing Kit (Cat. No. 4337455) was obtained from Life Technologies (Carlsbad, CA, USA). The ITS1 and ITS4 primers were synthesized by Invitrogen (Carlsbad, CA, USA).

UHPLC-HRMS analyses were performed using a Kromasil C18 column (250 × 4.6 mm, 5 μm; Cat. No. K08670357; Supelco/Merck, Gothenburg, VG, Sweden). For PGE_2_ and LTB_4_ quantification, an Agilent Poroshell 120 EC-C18 column (3.0 × 100 mm, 2.7 μm) coupled to an EC-C18 guard column (3.0 × 5 mm, 2.7 μm) was used (Agilent Technologies, Santa Clara, CA, USA).

### 4.2. Endophytic Fungi Isolation and Identification

The endophytic fungi evaluated in this study (OD1, OD5, OD6, OD7, and OD8) were isolated from the leaves of *O. diospyrifolia*, collected in June 2016 from an Atlantic Forest area in Alfenas, Minas Gerais, Brazil (W 46°07′29.2″ S 21°25′23.1″). The study was properly recorded in the National System for the Management of Genetic Heritage and Associated Traditional Knowledge (SisGen) under the registration code A913C3F. Botanist João Pedro Costa Elias authenticated the plant material, and a representative voucher specimen (UALF 2965) is currently at the Herbarium of the Federal University of Alfenas. The isolation of endophytic fungi was conducted following the methodology previously reported [38].

To accurately identify the most pharmacologically and chemically promising fungal isolate, molecular characterization of the Internal Transcribed Spacer (ITS) region was performed. Total genomic DNA was recovered following the mechanical disruption of the mycelial mass utilizing 425–600 μm glass beads [39]. PCR amplification of the target region was achieved using the standard primer pair ITS1 (5′-CCG TAG GTG AAC CTG CGG-3′) and ITS4 (5′-TCC TCC GCT TAT TGA TAT GC-3′) [40]. Sanger sequencing of the resulting amplicons was executed on an ABI 3500 XL Genetic Analyzer employing the BigDye Terminator chemistry (Cat. No. 4406016, Applied Biosystems, Foster City, CA, USA).

Sequence contig assembly was conducted using BioEdit software v. 7.7.1 [41]. Taxonomic proximity was determined by querying the assembled sequences against the NCBI GenBank database via the BLAST algorithm version 2.17.0+ [42]. For phylogenetic placement, the sequence dataset was aligned utilizing CLUSTALX v. 2.1 [43]. Evolutionary relationship was inferred in MEGA 11 [44] by applying the Neighbor-Joining method coupled with the Kimura 2-parameter substitution model [45]. The topological robustness of the phylogenetic tree was statistically evaluated through 1000 bootstrap iterations [46].

The ITS sequence generated for the OD5 strain has been deposited in GenBank under accession number QB001075.

Endophyte identification was inferred from the phylogenetic analysis of the ITS sequencing. Thus, the taxonomic assignment is based on a single genetic marker and should be interpreted accordingly.

### 4.3. Microbial Cultures and Extraction

All endophytic fungi were initially cultured on Petri dishes containing 20 mL of PDA. Seven-day-old fungal cultures were used as inoculum for all subsequent fermentation experiments. Each fungal strain (10 plugs, 6 mm diameter) was separately cultivated in 250 mL Erlenmeyer flasks containing 100 mL of PDB. All fermentations were performed in three independent biological replicates and incubated at 28 °C for 10 days in a biochemical oxygen demand (BOD) incubator TE-371/240L (Tecnal, Piracicaba, SP, Brazil).

At the end of the fermentation period, the mycelia were separated by vacuum filtration, and the resulting culture broth was extracted with EtOAc. The organic phase was collected and evaporated to dryness under reduced pressure at 40 °C using an RV 10 digital rotary evaporator (IKA, Staufen, BW, Germany) coupled to an MPC 105 T vacuum pump (Welch, Ilmenau, TH, Germany).

### 4.4. UHPLC-HRMS Analysis

Metabolic profiling of the crude extracts (OD1, OD5, OD6, OD7, and OD8) was acquired via UHPLC-HRMS. Aliquots of 1.0 μL (prepared at 0.5 mg/mL) were injected into a Shimadzu UHPLC system (LC-40D X3 series, incorporating the CBM-40, DGU-40S, SIL-40C X3, and CTD-40S units) hyphenated to a Shimadzu LCMS-9050 Q-TOF mass spectrometer featuring an electrospray ionization (ESI) interface. Chromatographic resolution was achieved on a Kromasil C18 stationary phase (250 × 4.6 mm, 5.0 µm particle size) maintained at a constant 40 °C. The mobile phase comprised a water–methanol mixture, using a linear gradient from 5% to 100% MeOH over 25 min, with a flow rate of 0.6 mL/min.

Mass spectrometry conditions were optimized as follows: interface temperature set to 300 °C, drying gas at 10 mL/min, nebulizing gas (N_2_) at 3.0 L/min, and a capillary voltage of 4.0 kV. Preliminary evaluations encompassing both polarities revealed that the positive ionization mode (*m*/*z* 100–1200) yielded superior analytical reproducibility, heightened signal-to-noise ratios, and a more comprehensive metabolic fingerprint for the endophyte EtOAc extracts. Consequently, only positive ion data were selected for the subsequent metabolomic assessments. MS1 data acquisition was performed with an event time of 0.100 s and a signal intensity threshold of 3000, with a mass range of 100–1200 *m*/*z*. Fragmentation was performed via data-independent acquisition (DIA) with a precursor signal intensity of 3000, divided into 32 fragmentation events with a Q1 resolution window of 34.4 and an event time of 0.034 s, totaling a loop time of 1.188 s. Argon was used as the gas in the collision cell, which operated at a collision energy (CE) of 40 and a spread of 10 (30–50). Product ions with *m*/*z* values between 50 and 1200 were detected. The samples were randomly analyzed.

Analytical blank consisting of ethyl acetate extract of the uninoculated culture medium was included in the UHPLC-HRMS analysis. The blank was used to identify and distinguish medium- and extraction-derived signals from those associated with the fungal cultures. However, quality-control (QC) samples were not included in the analytical sequence. Therefore, analytical reproducibility was evaluated by comparing analytical replicates, which showed consistent chromatographic and MS features.

### 4.5. Mass Data Processing and Dereplication

Raw spectrometric data acquired in the positive ion mode were imported into MZmine version 4.5.0 for comprehensive metabolomic feature processing. Prior to automated deconvolution, all total ion chromatograms (TIC) underwent visual inspection to ensure consistent run-to-run reproducibility. The data treatment pipeline involved centroiding, isotope filtering, peak alignment, blank subtraction, and missing value imputation.

Specific data-processing thresholds were established with mass detection noise levels at 1.5E7 (MS1) and 1.0E4 (MS2). The ADAP chromatogram builder required a minimum of 5 consecutive scans, an intensity cutoff of 4.5E7, an absolute peak height of 9.0E7, and an *m*/*z* tolerance of 0.002 (10.0 ppm). Feature deconvolution employed the local minimum resolver utilizing a 0.85 chromatographic threshold, a 0.05 RT/Mobility absolute search range, a minimum height of 9.0E5, a peak top-to-edge ratio of 2.0, a 0.06 min retention time filter, and an *m*/*z* tolerance of 0.01 (10.0 ppm). Isotopic signals were filtered based on a 3.0 ppm (0.01 *m*/*z*) window, a 0.02 min RT deviation, and a maximum charge state of 2, retaining the most intense isotope. Peak alignment was accomplished via the Join aligner algorithm, utilizing weighting factors of 3.0 for *m*/*z* (5.0 ppm tolerance) and 1.0 for retention time (0.06 min tolerance). Finally, gaps were filled, allowing a 5 ppm *m*/*z* variation, 20% intensity fluctuation, and 0.06 min RT shift across 3 minimum scans. Background features were computationally subtracted directly within the MZmine environment to yield a curated dataset free of blank interferences prior to downstream analysis.

Metabolite annotation was performed after data processing and feature detection. Molecular formulae were assigned according to the nitrogen rule, using a mass error tolerance of 5 ppm. MS/MS-based dereplication was subsequently performed on the SIRIUS 5 platform (Böcker Group/Bright Giant GmbH, Jena, Germany). Putative metabolite identities were prioritized by comparing the acquired tandem mass spectra with reference spectral data available in the platform databases. The highest-scoring candidates were selected as tentative annotations and further assessed based on their characteristic product ions and fragmentation patterns to support the proposed structures. Their fragmentation patterns were proposed based on diagnostic fragment ions and neutral losses (MS2 spectra).

To comply with established metabolomics standards, putative annotations of specialized metabolites were assigned with the confidence criteria established by the Metabolomics Standards Initiative [22].

### 4.6. Ex Vivo Anti-Inflammatory Assay—Quantification of PGE_2_ and LTB_4_ by UHPLC-MS/MS

The anti-inflammatory experiment was conducted on human blood, following previously reported methods [47]. The experiment was approved by the Research Ethics Committee of the Federal University of Alfenas (CAAE 60344622.1.0000.5142).

The ex vivo anti-inflammatory assay included randomized experiments evaluated in authentic experimental replicate. Pooled peripheral venous blood was obtained from two healthy volunteers aged 20 to 35 years. Blood was collected from donors who reported not having used any anti-inflammatory drugs for at least 15 days prior to blood collection. The blood was collected in vacuum tubes containing sodium heparin.

The experimental design included (i) unstimulated blood, (ii) blood stimulated with LPS (lipopolysaccharide, *Escherichia coli* O26) and a calcium ionophore to induce inflammation, (iii) vehicle-treated blood as the negative control, (iv) blood treated with fungal extracts, and (v) blood treated with reference drugs DEX and IND as the positive control. Negative control was used to determine the maximum levels of PGE_2_ and LTB_4_ produced under inflammatory conditions. Unstimulated blood samples and controls were used to assess the validity of the experiment and determine whether the observed effects were specifically associated with modulation of the stimulated production of PGE_2_ and LTB_4_.

The samples (fungal extracts) were resuspended in a solution of ethanol/water (1:1, *v*/*v*). The 1x phosphate-buffered saline (PBS; pH 7.2; 0.15 M chloride; 0.01 M phosphate) was used to prepare the dilutions of LPS or calcium ionophore solution. The positive controls were tested at a final well concentration of 1 μg/mL, and endophytic fungi extracts were evaluated at a final well concentration of 10 μg/mL. The LPS at 100 µg/mL (final well concentration) was used as the inflammatory inducer.

Anti-inflammatory biological evaluations were conducted in sterile 96-well microplates (86 × 128 mm) handled within a biological safety cabinet. The reaction mixtures consisted of 280 μL of heparinized whole blood, 35 μL of the evaluated extract, negative control, or standard drug, and 35 μL of either a calcium ionophore or LPS to trigger the inflammatory cascade. The total volume per well was fixed at 350 μL, ensuring the final vehicle concentration remained at an innocuous 10%. Microplates were incubated in a PHCbi incubator (MCO-170AIC, Tokyo, Japan), providing a humidified atmosphere of 5% CO_2_ at 37 °C for 24 h.

Following incubation, the plates were centrifugated (157 rcf, 4 °C, 5 min) in an Eppendorf 5424 R centrifuge to allow the recovery of 180 μL of plasma per well, which was subsequently frozen and lyophilized. To extract the analytes, the dried plasma residue was treated with 280 μL of a protein precipitation solvent (ACN/MeOH, 1:1, *v*/*v*) spiked with limaprost (23 ng/mL) as an internal standard. This mixture underwent vortexing, ultrasonic-assisted extraction for 2 min (Unique USC-1400), and high-speed centrifugation (4025 rcf, 4 °C, 10 min). The resulting supernatant (200 μL) was diluted in 1.8 mL of ultrapure water prior to solid-phase extraction (SPE). Strata LC-18 cartridges (100 mg) were pre-conditioned with methanol (2 mL) and 0.1% aqueous acetic acid (2 mL). Loaded samples were washed with 2 mL of the 0.1% acetic acid solution, eluted, and completely evaporated in a Thermo Fisher SpeedVac concentrator.

For the LC-MS/MS quantification of LTB_4_ and PGE_2_, the dried SPE eluates were dissolved in 100 μL of ACN. Chromatographic analysis was performed on a Shimadzu Prominence UPLC system (equipped with two LC-20 AD pumps, a DGU-20A3 degasser, SIL-20A HT autosampler, CTO-20A oven, and CBM-20A controller) coupled to an LC-8030 triple quadrupole mass spectrometer. Analyte separation occurred at 30 °C on an Agilent Poroshell 120 EC-C18 column (2.7 μm, 3 × 100 mm) protected by an Eclipse pre-column (1.8 μm, 2.1 × 5 mm), applying an injection volume of 20 μL. The mobile phase comprised 0.1% aqueous formic acid (A) and ACN (B), with a flow rate of 0.3 mL/min. The 8.5-min gradient profile started at 40% B, increased linearly to 100% B over 3 min, plateaued for 1 min, then rapidly returned to 40% B in 0.5 min, followed by a 4-min re-equilibration.

The mass spectrometer operated in negative ion mode under selected reaction monitoring, following a previously validated protocol [47]. Operating conditions included: nebulizing gas at 2 L/min, drying gas at 15 L/min (450 °C), interface voltage of 3.5 kV, DL temperature of 250 °C, detector voltage of 2.44 kV, and argon collision gas at 230 kPa. Data acquisition and peak integration were executed via LabSolutions software. Statistical variance among the triplicates was assessed utilizing one-way ANOVA paired with Dunnett’s multiple comparisons test in GraphPad Prism v. 9.1.2. Results were reported as mean ± standard deviation (SD), with statistical significance defined at *p*-values < 0.05.

## 5. Conclusions

The integrated biological and metabolomic workflow successfully pointed out the endophytic fungus *Lentinus* sp. OD5 as a promising producer of anti-inflammatory specialized metabolites among the isolates from *O. diospyrifolia*. *Lentinus* sp. OD5 crude extract displayed remarkable dual inhibition of the release of PGE_2_ and LTB_4_.

A comparative analysis of the chemical compositions of extracts from different endophytic fungi enabled the rapid prioritization of two metabolites from *Lentinus* sp. OD5, guiding downstream anti-inflammatory natural product discovery. High-resolution tandem mass spectrometry, combined with SIRIUS annotation, enabled the putative identification of these metabolites as tinocordiside and rhodojaponin II/V. The annotated fungal metabolites have been previously associated with anti-inflammatory activity through complementary mechanisms of action.

Overall, this study demonstrates that endophytic fungi from *O. diospyrifolia* are promising sources of anti-inflammatory specialized metabolites. These fungi, therefore, warrant further in-depth studies focused on the isolation and complete spectroscopic characterization of their metabolites, as well as investigations into their mechanisms of action.

## Figures and Tables

**Table 1:** Annotated specialized metabolites in the *Lentinus* sp. OD5 by UHPLC-HRMS.

No	Retention Time (min)	Theoretical/Observed Mass (Error ppm)	Putative Identification, Adduct	Molecular Formula
1	12.70 or 12.95	419.2040/419.2040 (0.00)	tinocordiside, [M+Na]^+^	C_21_H_32_O_7_
2	13.16	433.2197/433.2195 (−0.46)	rhodojaponin II/V, [M+Na]^+^	C_22_H_34_O_7_

## Data Availability

The data are contained within the article and Supplementary Materials.

## Supplementary Materials

The following supporting information can be downloaded at https://www.mdpi.com/article/10.3390/molecules31193499/s1: Table S1: Complete list of features detected in the evaluated endophytic fungi. Features exclusively detected in *Lentinus* sp. OD5 are highlighted in green, whereas putatively identified compounds are highlighted in red.
