# Supplementary material for: Dereplication of Natural Products from an Endophytic Fungus from Ocotea diospyrifolia with Anti-Inflammatory Potential Through Dual Modulation of PGE2 and LTB4 Release
Source: Molecules. 2026 Oct 1;31(19):3499. doi: 10.3390/molecules31193499 (PMC13666672; doi:10.3390/molecules31193499)
Supplement: Supplementary file 1 [file molecules-31-03499-s001.zip › molecules-4561576-supplementary.pdf]

Table S1. Complete list of features detected in the evaluated endophytic fungi. Features exclusively detected in *Lentinus* sp. OD5 are highlighted in green, whereas putatively identified compounds are highlighted in red.

ID	m/z	retention time	BLANK	OD5	OD6	OD8	OD2	OD4	OD7	QC
1	177,06602	3,1402	UNKNOWN	DETECTED	UNKNOWN	UNKNOWN	UNKNOWN	UNKNOWN	UNKNOWN	UNKNOWN
2	160,07583	3,4090667	UNKNOWN	DETECTED	UNKNOWN	UNKNOWN	UNKNOWN	UNKNOWN	UNKNOWN	UNKNOWN
3	145,08606	4,3671665	UNKNOWN	UNKNOWN	UNKNOWN	UNKNOWN	UNKNOWN	UNKNOWN	UNKNOWN	DETECTED
4	277,11855	4,38055	UNKNOWN	UNKNOWN	UNKNOWN	UNKNOWN	DETECTED	UNKNOWN	UNKNOWN	UNKNOWN
6	181,09728	4,82295	UNKNOWN	UNKNOWN	UNKNOWN	UNKNOWN	DETECTED	UNKNOWN	UNKNOWN	UNKNOWN
7	261,12363	4,9012065	UNKNOWN	DETECTED	UNKNOWN	UNKNOWN	DETECTED	DETECTED	DETECTED	DETECTED
8	283,10573	4,9014707	UNKNOWN	DETECTED	UNKNOWN	UNKNOWN	DETECTED	UNKNOWN	DETECTED	DETECTED
10	114,09149	4,9572835	UNKNOWN	UNKNOWN	UNKNOWN	UNKNOWN	UNKNOWN	UNKNOWN	DETECTED	UNKNOWN
11	146,06013	4,9838834	UNKNOWN	DETECTED	UNKNOWN	UNKNOWN	UNKNOWN	UNKNOWN	UNKNOWN	UNKNOWN
12	129,13867	5,0530667	UNKNOWN	UNKNOWN	UNKNOWN	UNKNOWN	DETECTED	UNKNOWN	UNKNOWN	UNKNOWN
13	223,18052	5,0530667	UNKNOWN	UNKNOWN	UNKNOWN	UNKNOWN	DETECTED	UNKNOWN	UNKNOWN	UNKNOWN
14	197,1286	5,2237053	UNKNOWN	DETECTED	UNKNOWN	DETECTED	DETECTED	DETECTED	DETECTED	DETECTED
15	229,15474	5,3412833	UNKNOWN	UNKNOWN	UNKNOWN	UNKNOWN	UNKNOWN	UNKNOWN	DETECTED	UNKNOWN
16	251,13663	5,3412833	UNKNOWN	UNKNOWN	UNKNOWN	UNKNOWN	UNKNOWN	UNKNOWN	DETECTED	UNKNOWN
17	147,0554	5,4635835	UNKNOWN	DETECTED	UNKNOWN	UNKNOWN	UNKNOWN	UNKNOWN	UNKNOWN	UNKNOWN
18	249,07334	5,5786667	UNKNOWN	DETECTED	UNKNOWN	UNKNOWN	UNKNOWN	UNKNOWN	UNKNOWN	UNKNOWN
19	224,05538	5,5906334	UNKNOWN	UNKNOWN	UNKNOWN	UNKNOWN	DETECTED	UNKNOWN	UNKNOWN	UNKNOWN
20	357,09693	5,6484833	UNKNOWN	UNKNOWN	UNKNOWN	UNKNOWN	UNKNOWN	UNKNOWN	DETECTED	UNKNOWN
21	185,12863	5,7061334	UNKNOWN	UNKNOWN	UNKNOWN	UNKNOWN	UNKNOWN	UNKNOWN	DETECTED	UNKNOWN
22	259,18074	5,8401165	UNKNOWN	UNKNOWN	UNKNOWN	UNKNOWN	DETECTED	UNKNOWN	UNKNOWN	UNKNOWN
23	211,14422	5,8981166	UNKNOWN	UNKNOWN	UNKNOWN	UNKNOWN	UNKNOWN	UNKNOWN	DETECTED	UNKNOWN
24	212,14709	5,9683166	UNKNOWN	DETECTED	UNKNOWN	UNKNOWN	DETECTED	UNKNOWN	UNKNOWN	UNKNOWN
25	211,14419	5,9696693	UNKNOWN	DETECTED	UNKNOWN	DETECTED	DETECTED	DETECTED	DETECTED	DETECTED

26	233,126	5,9779253	UNKNOWN	DETECTED	UNKNOWN	UNKNOWN	DETECTED	UNKNOWN	UNKNOWN	UNKNOWN
27	341,10216	6,05155	UNKNOWN	UNKNOWN	UNKNOWN	UNKNOWN	UNKNOWN	UNKNOWN	DETECTED	UNKNOWN
28	233,12605	6,073742	UNKNOWN	DETECTED	UNKNOWN	UNKNOWN	DETECTED	UNKNOWN	UNKNOWN	UNKNOWN
29	211,14423	6,0742373	UNKNOWN	DETECTED	UNKNOWN	DETECTED	DETECTED	UNKNOWN	UNKNOWN	DETECTED
30	245,1285	6,2258215	UNKNOWN	DETECTED	UNKNOWN	UNKNOWN	DETECTED	UNKNOWN	DETECTED	DETECTED
31	267,11062	6,230817	UNKNOWN	DETECTED	UNKNOWN	UNKNOWN	UNKNOWN	UNKNOWN	UNKNOWN	UNKNOWN
32	229,0973	6,31955	UNKNOWN	UNKNOWN	UNKNOWN	UNKNOWN	DETECTED	UNKNOWN	UNKNOWN	UNKNOWN
33	245,12853	6,357678	UNKNOWN	DETECTED	UNKNOWN	DETECTED	UNKNOWN	UNKNOWN	UNKNOWN	DETECTED
34	267,11064	6,3602753	UNKNOWN	DETECTED	UNKNOWN	UNKNOWN	UNKNOWN	UNKNOWN	UNKNOWN	DETECTED
35	246,13153	6,3651333	UNKNOWN	DETECTED	UNKNOWN	UNKNOWN	UNKNOWN	UNKNOWN	UNKNOWN	UNKNOWN
36	243,12039	6,749017	UNKNOWN	DETECTED	UNKNOWN	UNKNOWN	UNKNOWN	UNKNOWN	UNKNOWN	UNKNOWN
37	187,07551	7,1642666	UNKNOWN	UNKNOWN	UNKNOWN	UNKNOWN	DETECTED	UNKNOWN	UNKNOWN	UNKNOWN
39	343,12897	7,4329166	UNKNOWN	UNKNOWN	UNKNOWN	UNKNOWN	DETECTED	UNKNOWN	UNKNOWN	UNKNOWN
40	365,11075	7,4329166	UNKNOWN	UNKNOWN	UNKNOWN	UNKNOWN	DETECTED	UNKNOWN	UNKNOWN	UNKNOWN
41	685,25047	7,4329166	UNKNOWN	UNKNOWN	UNKNOWN	UNKNOWN	DETECTED	UNKNOWN	UNKNOWN	UNKNOWN
42	707,2323	7,4329166	UNKNOWN	UNKNOWN	UNKNOWN	UNKNOWN	DETECTED	UNKNOWN	UNKNOWN	UNKNOWN
44	285,07589	7,4782	UNKNOWN	DETECTED	UNKNOWN	UNKNOWN	UNKNOWN	UNKNOWN	UNKNOWN	UNKNOWN
45	527,28666	7,8373	UNKNOWN	UNKNOWN	UNKNOWN	UNKNOWN	UNKNOWN	UNKNOWN	DETECTED	UNKNOWN
46	293,13845	8,2968	UNKNOWN	UNKNOWN	UNKNOWN	UNKNOWN	DETECTED	UNKNOWN	UNKNOWN	UNKNOWN
47	315,12032	8,31595	UNKNOWN	UNKNOWN	UNKNOWN	UNKNOWN	DETECTED	UNKNOWN	UNKNOWN	UNKNOWN
48	289,07082	8,411834	UNKNOWN	UNKNOWN	UNKNOWN	UNKNOWN	DETECTED	UNKNOWN	UNKNOWN	UNKNOWN
49	225,10981	8,445689	UNKNOWN	UNKNOWN	DETECTED	UNKNOWN	UNKNOWN	DETECTED	UNKNOWN	DETECTED
50	219,06529	8,5077	UNKNOWN	UNKNOWN	UNKNOWN	UNKNOWN	DETECTED	UNKNOWN	UNKNOWN	UNKNOWN
51	389,1233	8,888733	UNKNOWN	UNKNOWN	DETECTED	UNKNOWN	UNKNOWN	UNKNOWN	UNKNOWN	UNKNOWN
52	411,10525	8,888733	UNKNOWN	UNKNOWN	DETECTED	UNKNOWN	UNKNOWN	UNKNOWN	UNKNOWN	UNKNOWN
53	245,0785	8,96355	UNKNOWN	DETECTED	DETECTED	UNKNOWN	UNKNOWN	UNKNOWN	UNKNOWN	DETECTED
54	149,02343	8,974733	UNKNOWN	DETECTED	UNKNOWN	UNKNOWN	UNKNOWN	UNKNOWN	UNKNOWN	UNKNOWN
55	409,21981	9,2433	UNKNOWN	DETECTED	UNKNOWN	UNKNOWN	UNKNOWN	UNKNOWN	UNKNOWN	UNKNOWN

56	277,17774	9,3289	UNKNOWN	UNKNOWN	UNKNOWN	UNKNOWN	UNKNOWN	UNKNOWN	DETECTED	DETECTED
57	295,18819	9,569467	UNKNOWN	DETECTED	UNKNOWN	UNKNOWN	UNKNOWN	UNKNOWN	UNKNOWN	UNKNOWN
58	511,29189	9,680067	UNKNOWN	UNKNOWN	UNKNOWN	UNKNOWN	UNKNOWN	UNKNOWN	DETECTED	UNKNOWN
59	239,12537	9,930685	DETECTED	DETECTED	DETECTED	DETECTED	DETECTED	DETECTED	DETECTED	DETECTED
61	307,18802	10,522966	UNKNOWN	UNKNOWN	UNKNOWN	UNKNOWN	DETECTED	UNKNOWN	UNKNOWN	UNKNOWN
62	255,15676	10,531748	DETECTED	DETECTED	DETECTED	DETECTED	DETECTED	DETECTED	DETECTED	DETECTED
63	277,17999	10,543683	UNKNOWN	UNKNOWN	UNKNOWN	UNKNOWN	UNKNOWN	UNKNOWN	DETECTED	UNKNOWN
64	317,17232	10,543683	UNKNOWN	UNKNOWN	UNKNOWN	UNKNOWN	UNKNOWN	UNKNOWN	DETECTED	UNKNOWN
65	530,24702	10,63815	UNKNOWN	UNKNOWN	UNKNOWN	UNKNOWN	DETECTED	UNKNOWN	UNKNOWN	UNKNOWN
66	552,2287	10,63815	UNKNOWN	UNKNOWN	UNKNOWN	UNKNOWN	DETECTED	UNKNOWN	UNKNOWN	UNKNOWN
67	391,20906	10,701167	UNKNOWN	DETECTED	UNKNOWN	UNKNOWN	UNKNOWN	UNKNOWN	UNKNOWN	UNKNOWN
68	530,24697	10,849667	UNKNOWN	UNKNOWN	UNKNOWN	UNKNOWN	DETECTED	UNKNOWN	UNKNOWN	UNKNOWN
69	552,22868	10,849667	UNKNOWN	UNKNOWN	UNKNOWN	UNKNOWN	DETECTED	UNKNOWN	UNKNOWN	UNKNOWN
70	274,27432	11,001033	UNKNOWN	DETECTED	DETECTED	DETECTED	DETECTED	DETECTED	DETECTED	DETECTED
71	318,30031	11,046417	UNKNOWN	UNKNOWN	UNKNOWN	UNKNOWN	DETECTED	UNKNOWN	DETECTED	DETECTED
72	362,32654	11,0737	UNKNOWN	UNKNOWN	UNKNOWN	UNKNOWN	UNKNOWN	UNKNOWN	UNKNOWN	DETECTED
75	230,24799	11,238533	UNKNOWN	UNKNOWN	DETECTED	UNKNOWN	UNKNOWN	UNKNOWN	UNKNOWN	UNKNOWN
77	277,17999	11,96335	UNKNOWN	UNKNOWN	UNKNOWN	UNKNOWN	UNKNOWN	UNKNOWN	DETECTED	UNKNOWN
78	299,16178	11,96335	UNKNOWN	UNKNOWN	UNKNOWN	UNKNOWN	UNKNOWN	UNKNOWN	DETECTED	UNKNOWN
79	301,14107	12,550836	DETECTED	DETECTED	DETECTED	DETECTED	DETECTED	DETECTED	DETECTED	DETECTED
80	149,02337	12,553227	DETECTED	DETECTED	DETECTED	DETECTED	DETECTED	DETECTED	DETECTED	DETECTED
81	279,15923	12,554286	UNKNOWN	DETECTED	DETECTED	DETECTED	UNKNOWN	DETECTED	DETECTED	DETECTED
82	205,19512	12,695817	UNKNOWN	DETECTED	UNKNOWN	UNKNOWN	UNKNOWN	UNKNOWN	UNKNOWN	UNKNOWN
83	414,24907	12,695817	UNKNOWN	DETECTED	UNKNOWN	UNKNOWN	UNKNOWN	UNKNOWN	UNKNOWN	UNKNOWN
84	815,41852	12,695817	UNKNOWN	DETECTED	UNKNOWN	UNKNOWN	UNKNOWN	UNKNOWN	UNKNOWN	UNKNOWN
85	419,20413	12,699224	UNKNOWN	DETECTED	UNKNOWN	UNKNOWN	UNKNOWN	UNKNOWN	UNKNOWN	DETECTED
86	419,20408	12,9452915	UNKNOWN	DETECTED	UNKNOWN	UNKNOWN	UNKNOWN	UNKNOWN	UNKNOWN	DETECTED
87	205,1951	12,945666	UNKNOWN	DETECTED	UNKNOWN	UNKNOWN	UNKNOWN	UNKNOWN	UNKNOWN	UNKNOWN

88	414,24901	12,945666	UNKNOWN	DETECTED	UNKNOWN	UNKNOWN	UNKNOWN	UNKNOWN	UNKNOWN	UNKNOWN
89	815,41845	12,945666	UNKNOWN	DETECTED	UNKNOWN	UNKNOWN	UNKNOWN	UNKNOWN	UNKNOWN	UNKNOWN
90	315,15684	13,128333	UNKNOWN	UNKNOWN	UNKNOWN	DETECTED	UNKNOWN	UNKNOWN	UNKNOWN	DETECTED
91	428,26468	13,15685	UNKNOWN	DETECTED	UNKNOWN	UNKNOWN	UNKNOWN	UNKNOWN	UNKNOWN	UNKNOWN
92	433,21982	13,1577	UNKNOWN	DETECTED	UNKNOWN	UNKNOWN	UNKNOWN	UNKNOWN	UNKNOWN	DETECTED
93	205,19512	13,177	UNKNOWN	DETECTED	UNKNOWN	UNKNOWN	UNKNOWN	UNKNOWN	UNKNOWN	DETECTED
94	375,21423	13,205875	UNKNOWN	DETECTED	UNKNOWN	UNKNOWN	UNKNOWN	UNKNOWN	UNKNOWN	DETECTED
95	315,15673	13,27995	DETECTED	UNKNOWN	DETECTED	DETECTED	UNKNOWN	UNKNOWN	UNKNOWN	DETECTED
96	284,33139	13,343567	UNKNOWN	UNKNOWN	UNKNOWN	UNKNOWN	DETECTED	UNKNOWN	UNKNOWN	UNKNOWN
97	353,26634	13,4051285	UNKNOWN	DETECTED	DETECTED	DETECTED	UNKNOWN	UNKNOWN	UNKNOWN	DETECTED
98	205,19519	13,4066	UNKNOWN	DETECTED	UNKNOWN	UNKNOWN	UNKNOWN	UNKNOWN	UNKNOWN	UNKNOWN
99	433,21992	13,4066	UNKNOWN	DETECTED	UNKNOWN	UNKNOWN	UNKNOWN	UNKNOWN	UNKNOWN	UNKNOWN
100	205,19515	13,483334	UNKNOWN	DETECTED	UNKNOWN	UNKNOWN	UNKNOWN	UNKNOWN	UNKNOWN	UNKNOWN
101	375,21426	13,5025	UNKNOWN	DETECTED	UNKNOWN	UNKNOWN	UNKNOWN	UNKNOWN	UNKNOWN	UNKNOWN
102	566,42798	13,532117	UNKNOWN	UNKNOWN	UNKNOWN	UNKNOWN	UNKNOWN	UNKNOWN	UNKNOWN	DETECTED
103	588,40978	13,589833	UNKNOWN	UNKNOWN	UNKNOWN	UNKNOWN	UNKNOWN	UNKNOWN	UNKNOWN	DETECTED
104	353,26632	13,676287	DETECTED	DETECTED	DETECTED	DETECTED	DETECTED	DETECTED	DETECTED	DETECTED
105	389,22995	13,809383	UNKNOWN	DETECTED	UNKNOWN	UNKNOWN	UNKNOWN	UNKNOWN	UNKNOWN	UNKNOWN
106	388,39379	13,923585	DETECTED	DETECTED	DETECTED	DETECTED	DETECTED	DETECTED	DETECTED	DETECTED
107	365,26632	14,018467	DETECTED	DETECTED	DETECTED	DETECTED	DETECTED	DETECTED	DETECTED	DETECTED
108	367,28191	14,144682	DETECTED	DETECTED	DETECTED	DETECTED	DETECTED	DETECTED	DETECTED	DETECTED
109	325,23503	14,149217	UNKNOWN	UNKNOWN	UNKNOWN	UNKNOWN	DETECTED	UNKNOWN	UNKNOWN	UNKNOWN
110	333,24	14,175869	DETECTED	DETECTED	DETECTED	DETECTED	DETECTED	DETECTED	DETECTED	DETECTED
111	335,25555	14,190252	DETECTED	DETECTED	DETECTED	DETECTED	DETECTED	DETECTED	DETECTED	DETECTED
112	335,25563	14,466257	DETECTED	DETECTED	DETECTED	UNKNOWN	DETECTED	DETECTED	DETECTED	DETECTED
113	377,26625	14,479471	DETECTED	DETECTED	DETECTED	DETECTED	DETECTED	DETECTED	DETECTED	DETECTED
114	353,26628	14,677592	DETECTED	DETECTED	DETECTED	DETECTED	DETECTED	DETECTED	DETECTED	DETECTED
115	379,28185	14,766656	DETECTED	DETECTED	DETECTED	DETECTED	DETECTED	DETECTED	DETECTED	DETECTED

116	313,27366	14,814737	UNKNOWN	DETECTED	DETECTED	DETECTED	DETECTED	DETECTED	DETECTED	DETECTED
117	683,54321	14,8159895	UNKNOWN	UNKNOWN	DETECTED	UNKNOWN	DETECTED	UNKNOWN	DETECTED	UNKNOWN
118	353,26624	14,81614	DETECTED	DETECTED	DETECTED	DETECTED	DETECTED	DETECTED	DETECTED	DETECTED
119	331,28424	14,81749	UNKNOWN	DETECTED	DETECTED	DETECTED	DETECTED	DETECTED	DETECTED	DETECTED
120	379,28192	14,914502	DETECTED	DETECTED	DETECTED	DETECTED	DETECTED	DETECTED	DETECTED	DETECTED
121	537,39492	15,281767	UNKNOWN	UNKNOWN	UNKNOWN	UNKNOWN	UNKNOWN	UNKNOWN	DETECTED	UNKNOWN
122	413,26624	15,370937	DETECTED	DETECTED	DETECTED	DETECTED	DETECTED	DETECTED	DETECTED	DETECTED
123	149,02338	15,372574	UNKNOWN	UNKNOWN	UNKNOWN	DETECTED	DETECTED	DETECTED	UNKNOWN	DETECTED
124	391,28424	15,373337	DETECTED	DETECTED	DETECTED	DETECTED	DETECTED	DETECTED	DETECTED	DETECTED
125	522,59737	15,537331	UNKNOWN	DETECTED	DETECTED	UNKNOWN	DETECTED	DETECTED	DETECTED	DETECTED
126	381,29754	15,570732	UNKNOWN	DETECTED	DETECTED	DETECTED	DETECTED	DETECTED	DETECTED	DETECTED
127	359,31555	15,572661	UNKNOWN	DETECTED	DETECTED	UNKNOWN	UNKNOWN	DETECTED	UNKNOWN	UNKNOWN
128	317,2452	15,762019	UNKNOWN	UNKNOWN	DETECTED	DETECTED	DETECTED	UNKNOWN	DETECTED	DETECTED
129	441,29766	15,943489	DETECTED	DETECTED	DETECTED	DETECTED	DETECTED	DETECTED	DETECTED	DETECTED
130	550,62864	16,112207	UNKNOWN	DETECTED	DETECTED	DETECTED	DETECTED	DETECTED	DETECTED	DETECTED
131	553,40758	16,160078	DETECTED	DETECTED	DETECTED	DETECTED	DETECTED	DETECTED	DETECTED	DETECTED
132	360,32362	16,336061	UNKNOWN	DETECTED	DETECTED	DETECTED	DETECTED	DETECTED	DETECTED	DETECTED
133	675,67603	16,336033	UNKNOWN	UNKNOWN	DETECTED	UNKNOWN	DETECTED	DETECTED	UNKNOWN	UNKNOWN
134	338,34173	16,355228	UNKNOWN	DETECTED	DETECTED	DETECTED	DETECTED	DETECTED	DETECTED	DETECTED
135	447,34684	16,687334	DETECTED	UNKNOWN	UNKNOWN	UNKNOWN	UNKNOWN	UNKNOWN	UNKNOWN	UNKNOWN
136	469,32901	16,715582	DETECTED	DETECTED	DETECTED	DETECTED	DETECTED	DETECTED	DETECTED	DETECTED
137	450,36352	16,871582	UNKNOWN	DETECTED	UNKNOWN	DETECTED	UNKNOWN	UNKNOWN	UNKNOWN	UNKNOWN
138	449,36024	16,872967	UNKNOWN	DETECTED	DETECTED	DETECTED	UNKNOWN	UNKNOWN	DETECTED	DETECTED
140	531,40806	17,640833	UNKNOWN	UNKNOWN	UNKNOWN	UNKNOWN	DETECTED	UNKNOWN	UNKNOWN	UNKNOWN
141	553,38994	17,640833	UNKNOWN	UNKNOWN	UNKNOWN	UNKNOWN	DETECTED	UNKNOWN	UNKNOWN	UNKNOWN
144	663,45366	19,1395	UNKNOWN	UNKNOWN	UNKNOWN	UNKNOWN	UNKNOWN	UNKNOWN	DETECTED	UNKNOWN
145	685,43552	19,163973	UNKNOWN	DETECTED	DETECTED	DETECTED	DETECTED	UNKNOWN	DETECTED	DETECTED
146	685,4354	19,234833	UNKNOWN	UNKNOWN	UNKNOWN	UNKNOWN	UNKNOWN	DETECTED	UNKNOWN	UNKNOWN

147	203,05909	20,108418	UNKNOWN	DETECTED	UNKNOWN	UNKNOWN	UNKNOWN	DETECTED	UNKNOWN	UNKNOWN
148	116,986	20,196335	UNKNOWN	UNKNOWN	DETECTED	UNKNOWN	UNKNOWN	UNKNOWN	UNKNOWN	DETECTED
149	203,05908	20,196695	DETECTED	UNKNOWN	DETECTED	DETECTED	DETECTED	UNKNOWN	DETECTED	DETECTED
150	149,01224	20,210445	DETECTED	DETECTED	UNKNOWN	DETECTED	UNKNOWN	DETECTED	DETECTED	DETECTED
151	116,98598	20,25675	DETECTED	DETECTED	UNKNOWN	DETECTED	DETECTED	DETECTED	DETECTED	UNKNOWN
152	149,01227	20,306667	UNKNOWN	UNKNOWN	DETECTED	UNKNOWN	DETECTED	UNKNOWN	UNKNOWN	UNKNOWN
